# A Rare Case of Sternoclavicular Tuberculosis Diagnosed Using Microchip-Based Polymerase Chain Reaction in a Diabetic Female

**DOI:** 10.7759/cureus.18845

**Published:** 2021-10-17

**Authors:** Subodh Kumar, Aroop Mohanty, Vivek Hada, Devesh P Singh, Garima Kushwaha

**Affiliations:** 1 Pulmonary Medicine, All India Institute of Medical Science Gorakhpur, Gorakhpur, IND; 2 Clinical Microbiology, All India Institute of Medical Science Gorakhpur, Gorakhpur, IND

**Keywords:** genexpert, truenat, ziehl neelsen, diabetes, sternoclavicular tb

## Abstract

Osteoarticular tuberculosis (TB) accounts for almost 10% of all extrapulmonary TB cases. In the majority of cases, the spine, knee, hip, and large bones are involved; other sites like the sternoclavicular joint, elbow, wrist, and smaller joints are infrequently involved. Uncommon locations of extrapulmonary TB pose a challenge in diagnosis due to lack of clinical suspicion, non-availability of samples, and unavailability of suitable diagnostic modalities. Here we report a case of uncommon location of osteoarticular TB diagnosed through microchip-based real-time polymerase chain reaction (PCR).

## Introduction

India has set itself an ambitious target for eliminating tuberculosis (TB) by the year 2025, five years earlier than the global target [1]. The modalities for the diagnosis of TB have changed drastically with the recent availability of molecular tests for routine diagnosis. The availability of various molecular testing platforms like GeneXpert, TrueNat, etc. is revolutionizing TB diagnostics and control programs [2]. Here we report a case of uncommon location of osteoarticular TB diagnosed using Truenat MTB.

## Case presentation

A 55-year-old female patient, with a history of treated pulmonary sarcoidosis, visited the Pulmonary Medicine outpatient department with complaints of fever and painful swelling in the lower part of the left side of the neck for two weeks. The swelling was insidious in onset and was gradually increasing in size. It was associated with pain, which was dull in nature and mild in intensity. The pain was aggravated with the movement of the neck and relieved with pain killers. The patient was suffering from steroid-induced diabetes mellitus, which was controlled on oral hypoglycemic agents.

On examination, she was febrile; pulse rate was 80 beats/min, respiratory rate was 22/min; blood pressure was 136/76mmHg; and oxygen saturation was 97% while breathing on room air. On local examination, there was a visible erythematous swelling of approximately 5 x 4 cm over the left sternoclavicular region. It was non-fluctuant and tender on palpation. There was no associated swelling present in the cervical and axillary areas. Auscultation of the chest revealed normal breath sounds without any crepitations. Rest systemic examination was normal. Routine laboratory investigations were performed and were found to be within normal limits.

**Figure 1:**
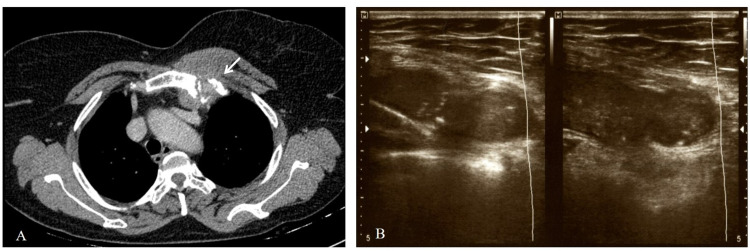
(A) CT scan of neck region showing (arrow) the necrotic destruction of left sternoclavicular joint with involvement of adjacent soft tissue. (B) USG showing the fluid-filled swelling with a needle in situ for aspiration.

Further, chest radiography was done that came out to be normal. In order to evaluate the swelling, a contrast-enhanced computerized tomography (CECT) thorax was performed. It revealed an inhomogeneous lesion involving the left sternoclavicular joint and adjacent first rib soft tissue component and mild fluid (Figure 1A). A USG-guided aspiration was performed from the swelling, but it was a dry tap. The minimal aspirate collected was sent for cytopathological examination and the patient was started on a broad-spectrum antibiotic (tablet amoxicillin/clavulanic acid 625 mg TDS) for seven days. Cytopathological examination revealed granulomatous inflammation with caseous necrosis. On follow-up after two weeks, the patient continued to be symptomatic, and an increase in the size of the swelling was noticed. USG of the swelling was performed which revealed a necrotic area with liquefaction (Figure 1B). A repeat USG-guided aspiration was performed immediately and the aspirated pus was sent to the Microbiology laboratory for detailed investigations. One part was used for staining and aerobic bacterial culture, whereas the other was used for TrueNat. Gram staining of the pus showed few pus cells with no microorganisms. On Ziehl-Neelsen staining, no acid-fast bacilli were seen. The bacterial culture also came out to be sterile after 48 hours. The other part of the aspirate was inoculated directly in lysis buffer provided with the Trueprep AUTO MTB Sample pretreatment pack (Molbio Diagnostics, Goa India). DNA was extracted using Trueprep Auto Universal Cartridge Based Sample Prep Device. Extracted DNA was added to the Truenat MTB microchip containing lyophilized master mix. The real-time polymerase chain reaction (PCR) was done using a pre-programmed profile on Truelab Quattro Real-Time Quantitative micro PCR Analyzer. It came out to be positive for Mycobacterium tuberculosis. Additionally, Rifampicin resistance was not detected in the sample using the Truenat MTB-RIF Dx kit. The reserved sample was sent for GeneXpert, which again confirmed the findings of Truenat. As a result, the patient was started on anti-tubercular therapy. On a follow-up visit, she became afebrile with a gradual decrease in the swelling size.

## Discussion

TB is an infectious disease caused by the airborne transmission of acid-fast bacillus *Mycobacterium tuberculosis. *It is a major cause of morbidity and mortality worldwide, especially in Southeast Asia. Extrapulmonary TB is commonly reported in forms of lymphadenitis, pleural effusion, osteoarticular and abdominal TB. Sternoclavicular joint TB is reported only in 1-3% of all osteoarticular TB and is one of the unusual locations of extrapulmonary TB [3]. The exact mechanism for primary sternal TB is still uncertain. It usually occurs due to a hematogenous spread from a pulmonary focus or a contiguous spread from apical pulmonary tuberculous focus to the joint. It may also be seen in immunocompromised conditions where following reactivation the organism may reach the joint via blood. In a retrospective analysis of 926 osteoarticular TB, nine cases of sternoclavicular TB were found over a period of five years [4]. The diagnosis in most of the cases was difficult due to the paucibacillary nature of the disease with the acid-fast bacilli being observed only in very few cases [3-5]. In our case also acid-fast bacilli were not observed in microscopy, although the granulomatous inflammation was seen in the cytological examination after fine-needle aspiration. Due to past history of sarcoidosis, the cytopathological report was not strong enough evidence for starting anti-tubercular therapy in our case. Availability of Truenat helped in arriving at the diagnosis in our case. Truenat MTB is an indigenous molecular test that has been recently endorsed by the WHO for the diagnosis of pulmonary TB as a first-line test. The ability to detect rifampicin resistance using the same platform is a distinct advantage provided by this platform over direct microscopy [6]. In the study conducted on the sputum samples, it was seen that Truenat MTB results were largely concordant with other WHO-endorsed nucleic acid amplification test platforms, GeneXpert. The Truenat MTB had good sensitivity and specificity in case of detection with hands-on time of fewer than three hours as well as fits the requirements in resource-limited health care settings [7]. During the current COVID-19 pandemic, Truenat was one tool that was utilized for the diagnosis of severe acute respiratory syndrome coronavirus 2 (SARS-CoV-2) infection in India with more than 2500 Truelab workstations currently operational at 1008 sites in 530 districts of India [8]. This technological infrastructure built for the COVID-19 pandemic may be leveraged for ramping up the diagnosis of other major diseases like TB, HIV, etc [9].

## Conclusions

Sternal TB is usually misdiagnosed as osteoarthritis or inflammatory arthritis or even as a traumatic event. Various diagnostic modalities compatible with resource-limited settings are now available for microbiological confirmation of TB. Truenat is one such platform that has been approved for diagnosing pulmonary TB. Its use in detecting extrapulmonary TB will provide the necessary boost to the national TB elimination program. This case highlights the need to evaluate the use of the Truenat platform in the diagnosis of extrapulmonary TB.
